# Differential expression of Cathepsin E in transthyretin amyloidosis: from neuropathology to the immune system

**DOI:** 10.1186/s12974-017-0891-9

**Published:** 2017-06-06

**Authors:** Nádia Pereira Gonçalves, João Moreira, Diana Martins, Paulo Vieira, Laura Obici, Giampaolo Merlini, Margarida Saraiva, Maria João Saraiva

**Affiliations:** 1 i3S – Instituto de Investigação e Inovação em Saúde da Universidade do Porto, Rua Alfredo Allen, 208, 4200-135 Porto, Portugal; 2 Molecular Neurobiology Group, IBMC – Institute for Molecular and Cell Biology, University of Porto, 4150-180 Porto, Portugal; 3 Unité du Développement des Lymphocytes, Département d’Immunologie, Institut Pasteur, Paris, 75724 CEDEX 15 France; 4 Amyloidosis Research and Treatment Center, Department of Molecular Medicine, Fondazione Instituto di Ricovero e Cura a Carattere Scientifico (IRCCS) Policlinico San Matteo, University of Pavia, Pavia, Italy; 5 Immune Regulation Group, IBMC – Institute for Molecular and Cell Biology, University of Porto, 4150-180 Porto, Portugal; 6 Present address: Department of Biomedicine/DANDRITE, Aarhus University, Ole Worms Alle 3, 1171, 8000 Aarhus C, Denmark

**Keywords:** Cathepsin E, Transthyretin, Immune regulation, Macrophages, Neurodegeneration, Familial amyloidotic polyneuropathy

## Abstract

**Background:**

Increasing evidence supports a key role for inflammation in the neurodegenerative process of familial amyloidotic polyneuropathy (FAP). While there seems to be an overactivation of the neuronal interleukin-1 signaling pathway, the immune response is apparently compromised in FAP. Accordingly, little immune cell infiltration is observed around pre-fibrillar or fibrillar amyloid deposits, with the underlying mechanism for this phenomenon remaining poorly understood. Cathepsin E (CtsE) is an important intermediate for antigen presentation and chemotaxis, but its role in the pathogenesis of FAP disease remains unknown.

**Methods:**

In this study, we used both mouse primary macrophages and in vivo studies based on transgenic models of FAP and human samples to characterize CtsE expression in different physiological systems.

**Results:**

We show that CtsE is critically decreased in bone marrow-derived macrophages from a FAP mouse model, possibly contributing for cell function impairment. Compromised levels of CtsE were also found in injured nerves of transgenic mice and, most importantly, in naïve peripheral nerves, sensory ganglia, murine stomach, and sural nerve biopsies derived from FAP patients. Expression of CtsE in tissues was associated with transthyretin (TTR) deposition and differentially regulated accordingly with the physiological system under study. Preventing deposition with a TTR small interfering RNA rescued CtsE in the peripheral nervous system (PNS). In contrast, the expression of CtsE increased in splenic cells (mainly monocytes) or peritoneal macrophages, indicating a differential macrophage phenotype.

**Conclusion:**

Altogether, our data highlights the potential of CtsE as a novel FAP biomarker and a possible modulator for innate immune cell chemotaxis to the disease most affected tissues—the peripheral nerve and the gastrointestinal tract.

**Electronic supplementary material:**

The online version of this article (doi:10.1186/s12974-017-0891-9) contains supplementary material, which is available to authorized users.

## Background

Transthyretin (TTR) variants are associated with hereditary amyloidoses, clinically termed familial amyloidotic polyneuropathy (FAP) or cardiomyopathy when point mutations in the *TTR* gene result in protein deposition in the peripheral nervous system (PNS) or heart, respectively [1].

TTR is a homotetrameric protein mainly synthesized by liver hepatocytes and the choroid plexuses of the brain [2, 3], acting as a transport protein for about 15% of circulating plasma thyroxine and almost all retinol [4]. In addition, during the last years, several other functions have been associated with this molecule, like its potential role in high-density lipoprotein biology [5], cognition [6], neuroprotection after cerebral ischemia [7] via a megalin dependent pathway [8], modulation of Aβ aggregation [9], regulation of 14-3-3ζ metabolism [10], or glucose homeostasis [11]. However, the existence of genetic point mutations may alter the quaternary structural stability of TTR [12], leading to its dissociation into non-native species which self-assemble and polymerize, ultimately depositing in the extracellular matrix and disrupting normal tissue function [13, 14].

Different studies evidence that low molecular mass aggregated species are the most cytotoxic culprits [15, 16] inducing cell apoptosis, oxidative stress, inflammation, extracellular matrix remodeling, endoplasmic reticulum stress, and activation of the heat shock response [17]. TTR-induced cytotoxicity was in part attributed to the binding of TTR species to the receptor for advanced glycation end products, with activation of extracellular signal-regulated kinases 1/2, nuclear factor κB, and consequent transcription of pro-inflammatory proteins [18–20]. Very recently, studies with human chondrocytes demonstrate that increased toll-like receptor-4 and p38 phosphorylation are signaling mechanisms involved in the induction of cytokines and oxidative stress intermediates by TTR, suggesting the importance of inflammation for FAP progression [21]. Importantly, although being consequent to mutant TTR tissue deposition, inflammation was also found to be a disease causing agent, triggering TTR deposition and neurodegeneration [19, 22–24]. Nevertheless, despite of this pro-inflammatory milieu, no immune cellular infiltrate is usually found near TTR deposits, which could be partially related to the impaired production of chemokines and neurotrophins by the Schwann cells [19, 23, 25].

Cathepsin E (CtsE) is an aspartic protease mainly located at the endosomal compartment but also found in the endoplasmic reticulum and Golgi complex in various cell types [26]. Its expression is highly upregulated in gastrointestinal and breast cancers, being thus considered as a potential biomarker in these situations [27, 28]. Despite the controversy around the clinical significance of the increased CtsE expression in carcinogenesis [26], it was found that CtsE has antitumorigenic properties by the specific cleavage of tumor necrosis factor-related apoptosis ligand at the surface of cancer cells, thereby inducing growth arrest and apoptosis in cancer cells without harming normal cells [29]. Moreover, CtsE might also play key roles in the process of neurodegeneration since the levels of this enzyme were found upregulated in brains from patients with Alzheimer’s disease [30] and CtsE activity was also increased in cerebrospinal fluid of Parkinson’s disease patients [31]. These observations together with the downregulated CtsE levels found in the peripheral nerve of a TTR V30M transgenic mouse model in previous microarrays [32] prompt us to investigate the potential involvement of CtsE in FAP pathogenesis by using tissues from FAP patients and different well-characterized mouse models of disease [23, 33, 34].

## Methods

### Ethics

All the animal procedures were performed according to the European Union directive (2010/63/EU) and approved by the local ethical committee rules (DL 113/2013).

All human samples were collected from donors whose written informed consent for the use of the material and clinical information for research purposes.

### Human samples

Sural nerve biopsy specimens from asymptomatic members of FAP kindred’s (*n* = 3), FAP patients (*n* = 3), and normal controls (*n* = 3) were kindly provided by the Hospital Geral de Santo António, Porto, Portugal. Amyloid deposits (assessed by Congo red staining) and nerve fiber density (morphometric studies) were previously characterized in the hospital, for disease stage characterization and obtained as part of the clinical diagnosis, before the current use of less invasive methods, as previously described [15]. Sural nerve biopsies from asymptomatic individuals had no amyloid deposition, and all these patients were asymptomatic at the time of biopsy (with no signs of neuropathy). No significant reduction in the number of nerve fibers was observed when compared to normal scores and no fiber degeneration was found, meaning that samples from asymptomatic carriers were distinguished from their non-carrier normal siblings by the presence of non-fibrilar TTR deposition. With time, these patients will eventually develop neuropathy and become symptomatic.

Twenty milliliters of blood samples from *n* = 5 V30M FAP patients (three males and two females), aged between 40 and 60 years old, and age- and gender-matched controls were collected to tubes containing EDTA. Written informed consent for use of biologic samples and clinical data for research purposes was provided according to the local institutional review board guidelines at Policlínico San Matteo, University of Pavia, Italy.

### Animals

Six-month-old male and female TTR wild-type (WT) and TTR V30M mice (null for the endogenous mouse *Ttr* gene), in the 129/Sv background, were used for the experiments with sciatic nerve injury (*n* = 5 per group).

Naïve V30M and WT animals (*n* = 6) having 9 months of age were used to evaluate tissues in a basal situation.

For TTR silencing studies, a different FAP mouse model (carrying the V30M mutation but in a heterozygous background for the heat shock factor 1 (*Hsf-1*)) was chosen due to the deposition of TTR non-fibrillar species in the peripheral nervous system.

For bone marrow-derived macrophages, 3-month-old V30M and WT mice (*n* = 9 each strain) were used.

Animals were housed in a controlled temperature room, maintained under a 12-h light/dark period, with water and food ad libitum and then sacrificed with a lethal injection of a premixed solution containing ketamine (75 mg/kg) plus medetomidine (1 mg/kg).

### Sciatic nerve injury

For sciatic nerve injury, 6-month-old WT (*n* = 5) and V30M (*n* = 5) mice were anesthetized (i.p. 100 mg/kg ketamine and 1 mg/kg medetomidine) and the exposed left sciatic nerve was injured with a single ligature at the mid-thigh level, adapted from the method of Brumovsky et al., [35], as previously described [23]. The sham contralateral nerve was open but not constricted and used as a control. Mice were kept in a recovery room with infrared heating lamp for 1–2 h, and post-surgical analgesia consisted of subcutaneous injections of butorphanol (1 mg/kg) twice a day for the first 2 days. Seven days later, injured sciatic nerves and ipsilateral L3–L5 dorsal root ganglia (DRG) were collected and processed for immunohistochemistry, real-time PCR, or western blot analysis.

### Liver TTR silencing in a FAP mouse model

TTR or control small interfering RNA (siRNA) (vehicle) were formulated into a lipid nanoparticle delivery system [36]. Six-month-old Hsf/V30M mice were injected in the tail vein with human TTR siRNA (*n* = 4), at a concentration of 1 mg/kg. Untreated age-matched controls received only the vehicle intravenously (*n* = 4). One injection per week was performed during 4 weeks, and animals were euthanized 48 h after the last injection, with a lethal anesthesia of ketamine/medetomidine. Lumbar DRG were immediately frozen at −80 °C in RNA latter (Ambion) for subsequent real-time PCR analysis.

### Bone marrow derived macrophages

For cell culture experiments, cRPMI medium (Roswell Park Memorial Institute) was prepared by supplementing RPMI 1640 (Lonza) with 10% heat-inactivated fetal bovine serum, 1% sodium pyruvate, 1% HEPES, 1% l-glutamine (GIBCO), and 0.05 mM 2-mercaptoethanol (Sigma-Aldrich). Bone marrow cells were flushed from the femurs and tibia of mice and plated at 4 × 10^6^ cells per petri dish (bacterial plates; Sterilin), in 8 mL of culture medium supplemented with 20% L929 cell-conditioned media (LCCM). By day 4, the cells were fed with 10 mL of the same media, and at day 6, macrophages were harvested and seeded into 24-well tissue culture plates at 0.5 × 10^6^ cells/mL. Cells were rested overnight and used for immunocytochemistry or western blot in the day after.

### Semi-quantitative Immunohistochemistry

For immunohistochemical analysis, mice sciatic nerves, DRG, spleen, and stomach (*n* = 6) were excised and post-fixed in 10% formalin at room temperature. Paraffin sections of human sural nerve biopsies were also used. After deparaffination (Histoclear, National Diagnostics) and tissue hydration in descent alcohol series, the sections were quenched for 30 min in 3% hydrogen peroxide in methanol. After extensive wash, slides were blocked with phosphate buffer supplemented with 10% fetal bovine serum and 0.5% Triton X-100 for 1 h and incubated overnight with the primary antibody against CtsE (1:100, rabbit polyclonal, Abcam). Biotinylated anti-rabbit (1:200; Vector) secondary antibody was incubated for 45 min at room temperature. Negative controls were included by omitting the primary or secondary antibodies, but these never yielded staining patterns. An amplification step was performed, using avidin-biotin-peroxidase complex (ABC Elite; Vector laboratories), followed by washing and incubation with 3.3′-diaminobenzidine (Sigma-Aldrich). Finally, tissue sections were counterstained with hematoxylin, and mounted and visualized in an optical microscope. For semi-quantitative analyses of the immunostained slide, five pictures at ×20 magnification were taken from different areas and quantified using the Image pro-plus 5.1 software (Media Cybernetics). Results shown represent the area occupied by pixels corresponding to the substrate reaction color that is normalized relatively to the total image area, with the corresponding standard error of the mean (SEM).

### Confocal microscopy

For mouse cell imaging by fluorescence analysis, cells were plated over a glass lipopolysaccharide-free coverslip, in a 24-well plate, and fixed with 4% paraformaldehyde. After permeabilization, fixed cells were blocked with phosphate buffer supplemented with 10% fetal bovine serum and 0.5% Triton X-100 for 1 h at room temperature and incubated overnight at 4 °C with rabbit polyclonal antibody against CtsE (1:100, Abcam) or a rat monoclonal anti-F4/80 antibody (1:100, Serotec), as a macrophage marker.

For WT mouse tissue imaging, the sciatic nerve and stomach (*n* = 3) were processed as described above. After deparaffinazation, sections were hydrated and permeabilized with 0.5% Triton X-100 for 10 min and then blocked with the previous mentioned blocking solution for 1 h at room temperature. After blocking, tissues were incubated overnight at 4 °C with the primary antibodies: rabbit polyclonal anti-CtsE (1:100, Abcam), mouse monoclonal anti-βIII-tubulin (1:1000, Promega), goat polyclonal anti-ionized calcium-binding adaptor molecule 1 (IBA-1, 1:100), mouse monoclonal anti-S100 (1:50, Abcam), and/or potassium-transporting ATPase subunit beta (Atp4b, 1:100).

Secondary antibodies included goat anti-mouse Alexa Fluor 568, donkey anti-rabbit Alexa Fluor 488, donkey anti-goat, or anti-rat Alexa Fluor 568 (1:1000, Molecular Probes). Sections were washed and cover-slipped in Vectashield, including DAPI as a nuclear dye (Vector Laboratories) and visualized under a Laser Scanning Confocal microscope Leica SP5.

### Messenger RNA isolation, cDNA synthesis, and real-time quantitative polymerase chain reaction

Messenger RNA (mRNA) from mice spleen, bone marrow, cultured macrophages and human blood were isolated by phenol extraction (Trizol, Invitrogen). Sciatic nerves (*n* = 6 per group) were dissected free from surrounded tissues and frozen in RNA later (Ambion) for subsequent RNA purification using RNeasy Mini columns (Qiagen), according to the manufacturer’s guidelines. First-strand complementary DNA (cDNA) was synthesized using the SuperScript double-stranded cDNA Kit (Invitrogen) and quantitative real-time PCR performed using the iQ Syber Green Super Mix (Bio-Rad). Samples were run in duplicate and results analyzed by the Bio-Rad iQ5 software. Glyceraldehyde 3-phosphate dehydrogenase (*Gapdh*) was used as reference gene. For the quantification of mRNA expression levels, the reaction was performed in a final volume of 20 μL containing 0.5 μL of each specific primer: mouse *Gapdh* forward: GCCTTCCGTGTTCCTACC, mouse *Gapdh* reverse: AGAGTGGGAGTTGCTGTTG; mouse *CtsE* forward: GCTATGACCCCTCTCATTTCT, mouse *CtsE* reverse: AGGTCCCTGTGTCCACTATG; human *GAPDH* forward: GAGTCCACTGGCGTCTTC, human *GAPDH* reverse: GATGATCTTGAGGCTGTTGTC; human *CTSE* forward: CTCTGAGTTCTGGAAATC, human *CTSE* reverse: AAGATGACAGTGAAGTTC, (all from Sigma), and 18 μL of Mix plus 1 μL of the newly synthesized cDNA. Primer sequences were designed using Beacon Designer 8 (Premier Biosoft) for all genes. Analysis of real-time PCR data was made by the comparative CT method. Individual relative gene expression values were calculated using the following formula: 2 ^−^^(Ct gene of interest − Ct constitutive gene)^.

### Flow cytometry

After mice anesthesia (*n* = 6 WT and *n* = 6 V30M mice), peritoneal cells, blood, and spleen were collected. Single cell suspensions of spleen were prepared in the flow chamber. Specifically, using the plunger end of the syringe, the tissue was mashed through the cell strainer to obtain single cell suspensions. Peritoneal cells were recovered by washing the abdominal cavity with PBS. Subsequently, cells were counted and centrifuged at 1500 rpm for 5 min at 4 °C and resuspended in staining buffer (eBioscience). Before extracellular staining, cells were incubated with anti-mouse CD16/CD32 (eBioscience) for Fc receptor blocking. Cells were then surface stained with APC eFluor® 780 anti-mouse F4/80 (clone BM8, 1:100, BioLegend), Pacific Blue anti-mouse CD11c (clone N418, 1:100, BioLegend), PE anti-mouse CD11b (clone M1/70, 1:100BioLegend), PerCP-Cyanine 5.5 MHC II (clone M5/114.15.2, 1:100, BioLegend), APC anti-mouse Ly6G (clone 1A8, 1:100, BioLegend), and PE-Cy7 anti-mouse Ly6C (clone HK1.4, 1:100, BioLegend). Thereafter, cells were fixed and permeabilized (Foxp3 fixation/permeabilization buffer, eBioscience) for intracellular staining with FITC anti-mouse CtsE (1:100, Abcam) or the respective isotype control. Data acquisition was performed in a FACSCantoTM II system (BD Biosciences) using the FACSDIVATM software (BD) and compensated and analyzed in FLOWJO version 9.7.5. (Tree Star Inc.).

### Western blot analysis

Protein was isolated from murine-injured sciatic nerve or cultured macrophages by homogenization with lysis buffer containing 5 mM ethylenediamine tetraacetic acid, 2 mM ethylene glycol tetraacetic acid, 20 mM 3-(*N*-morpholino) propanesulfonic acid, 0.5% Triton X-100, 30 mM sodium fluoride, 40 mM sodium pyrophosphate, 1 mM sodium orthovanadate, and 1 mM phenylmethylsulphonyl fluoride, supplemented with a protease inhibitor cocktail (GE Healthcare). Protein concentration was determined using the Bradford protein assay (Bio-Rad). Subsequently, samples were run on a 12% polyacrylamide gel electrophoresis (SDS-PAGE) and blotted on nitrocellulose WhatmanTM membrane (GE Healthcare). Primary antibodies against CtsE and GAPDH were diluted in 5% bovine serum albumin in PBS supplemented with 0.1% Tween 20 (PBS-T), overnight at 4 °C. In the next day, blots were washed with PBS-T and incubated with anti-rabbit conjugated to horseradish peroxidase (1:5,000; The Binding Site) for 45 min at room temperature. After three washes in PBS-T, bands were visualized with enhanced chemiluminescence using the LuminataTM Crescendo (Millipore). Protein bands were quantified by densitometry using Quantity One software (Bio-Rad), and GAPDH quantification was used to correct for total protein loading variation.

### Statistical analysis

Statistical comparison of data was performed using the Student *t* test or one-way ANOVA with Graph Pad Prism software. Quantitative data are expressed as mean ± SEM. Statistical significance was established for p* < 0.05, p** < 0.01, and p*** < 0.001.

## Results

### Expression of Cathepsin E is downregulated in injured nerves from V30M animals

**Fig. 1:**
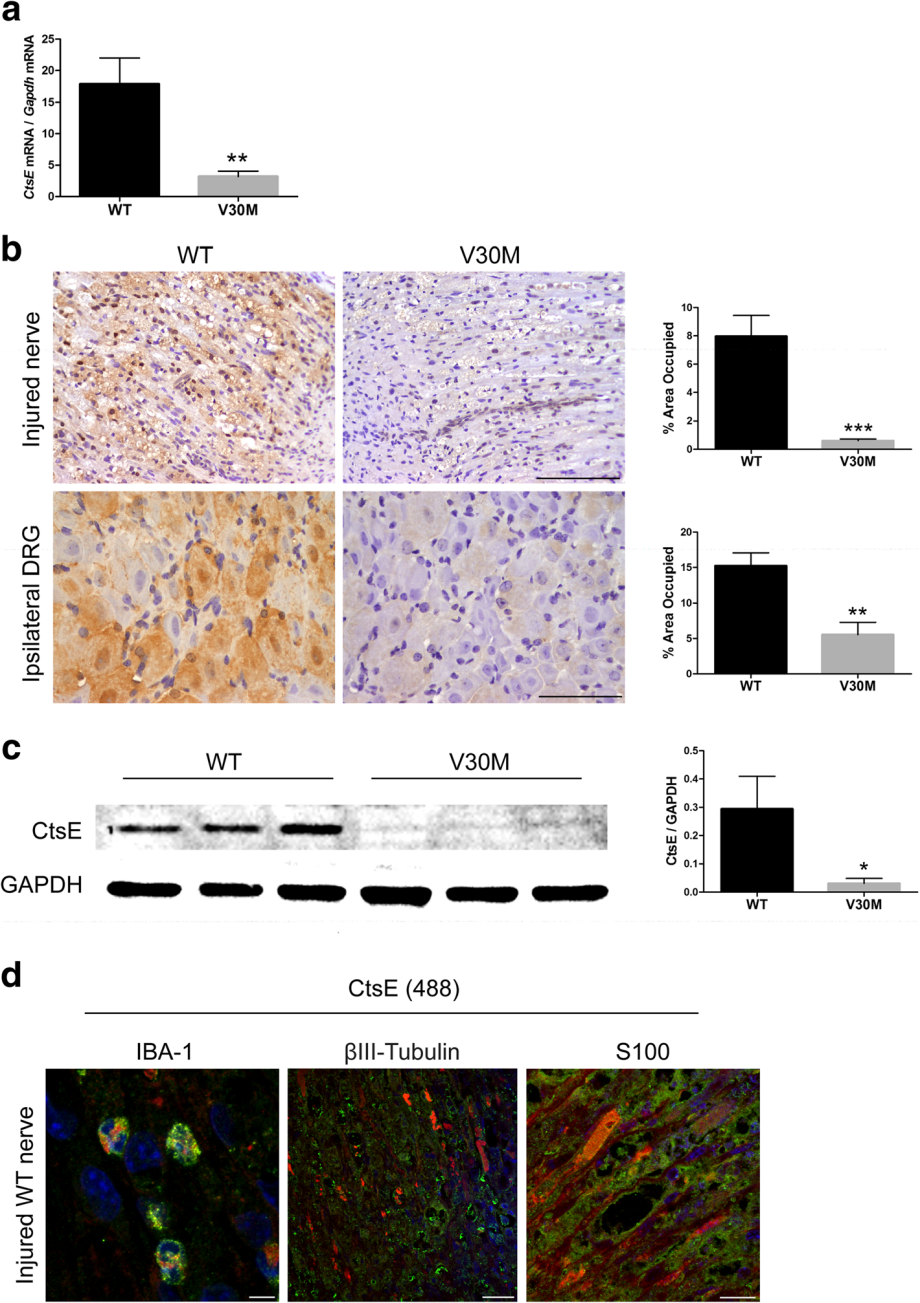
Sciatic nerve injury leads to PNS downregulation of CtsE expression in V30M mice. **a** Histogram represents *CtsE* mRNA levels in the sciatic nerve of WT and V30M mice, 7 days after injury (*n* = 5 for each group/**p < 0.01). **b** Representative SQ-IHC against mouse CtsE in WT and V30M injured nerves (*upper panel*) and lumbar ipsilateral DRGs (*lower panel*). *Scale bar* 50 μm. *Charts* represent quantification of immunohistochemical images, and data is represented as mean ± SEM (***p < 0.001 and **p < 0.01). **c** Representative anti-CtsE western blot from injured WT nerves versus injured nerves from V30M mice. Histogram denotes normalized CtsE/Gapdh density quantification ± SEM (*p < 0.05). **d** Double immunofluorescence between CtsE (*green*) and IBA-1, BIII-Tubulin or S100 (all in *red*), denoting CtsE localized mainly in macrophages and Schwann cells (*yellow*/*orange*); ×20 magnification

We have previously performed a transcriptomic analysis of injured sciatic nerves from WT versus V30M mice and found a substantial decrease on the mRNA levels of *CtsE* in the transgenic group, with a fold change of 9.31 [32]. Considering this pronounced difference and the unknown role of CtsE in FAP, we decided to further investigate this finding. We started by validating the differences in CtsE expression by quantitative real-time PCR (qPCR). In line with our microarray data, *CtsE* expression in injured nerve was significantly downregulated in the FAP transgenic animal model when compared to WT (Fig. 1a). Furthermore, results from semi-quantitative immunohistochemistry (SQ-IHC) and western blot displayed a considerable decrease in the protein levels of CtsE in injured V30M nerves comparatively to WT, corroborating the genetic scenario (Fig. 1b, c). Interestingly, CtsE was also significantly reduced in sensory neurons ipsilateral to the site of injury in V30M animals (Fig. 1b).

To understand the source of CtsE in the nerve tissue after lesion, we evaluated the colocalization of CtsE with markers for neurons, Schwann cells, and macrophages by confocal microscopy. The findings support CtsE expression by activated macrophages and Schwann cells (Fig. 1d).

### CtsE is reduced in neurons and axons of the peripheral nervous system and in stomach of V30M naïve mice

**Fig. 2:**
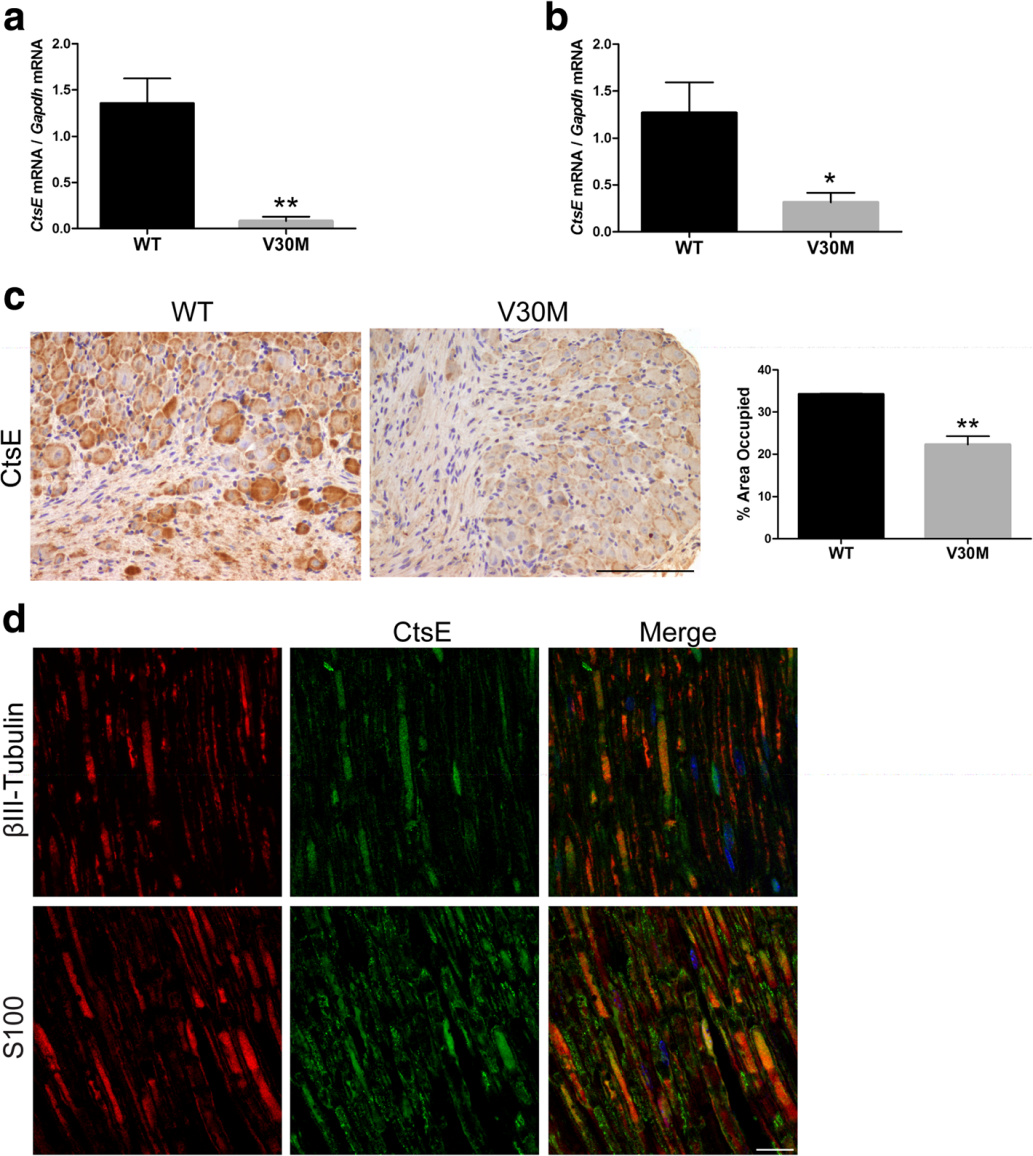
Comparative CtsE expression analyses in naïve WT and V30M mice. **a, b** Histograms represent *CtsE* mRNA levels in WT and V30M naïve sciatic nerves and DRGs, respectively (**p < 0.01, *p < 0.05). **c** Representative SQ-IHC against mouse CtsE in WT and V30M DRGs. Charts show quantification of immunohistochemical images, and data is represented as mean ± SEM (***p < 0.001, **p < 0.01). *Scale bar* 100 μm. **d** Double immunofluorescence of CtsE (in *green*), BIII-Tubulin, or S100 (all in *red*), showing that in an uninjured nerve, CtsE is localized in both peripheral axons and Schwann cells; ×20 magnification

Next, we wondered if the alterations in the transcriptional regulation of CtsE in the lesioned V30M peripheral nerve were also present in the naïve situation. To address this question, we compared the mRNA and protein levels of CtsE in naïve mice carrying the TTR V30M mutation to naïve WT animals. Real-time PCR revealed a significant downregulation of *CtsE* in nerves and DRG from the FAP mouse model comparatively with the control group (Fig. 2a, b). CtsE protein levels were also decreased in V30M DRG as determined by immunohistochemical analyses (Fig. 2c), although only a small trend was found regarding the sciatic nerve (data not shown). Additionally, studies with confocal microscopy of naïve WT nerves indicated CtsE colocalization with S100 and βIII-tubulin (Fig. 2d), supporting CtsE localization in both Schwann cells and peripheral axons. Overall, these results demonstrate, for the first time, an impaired production of this enzyme in the PNS of V30M mice.

**Fig. 3:**
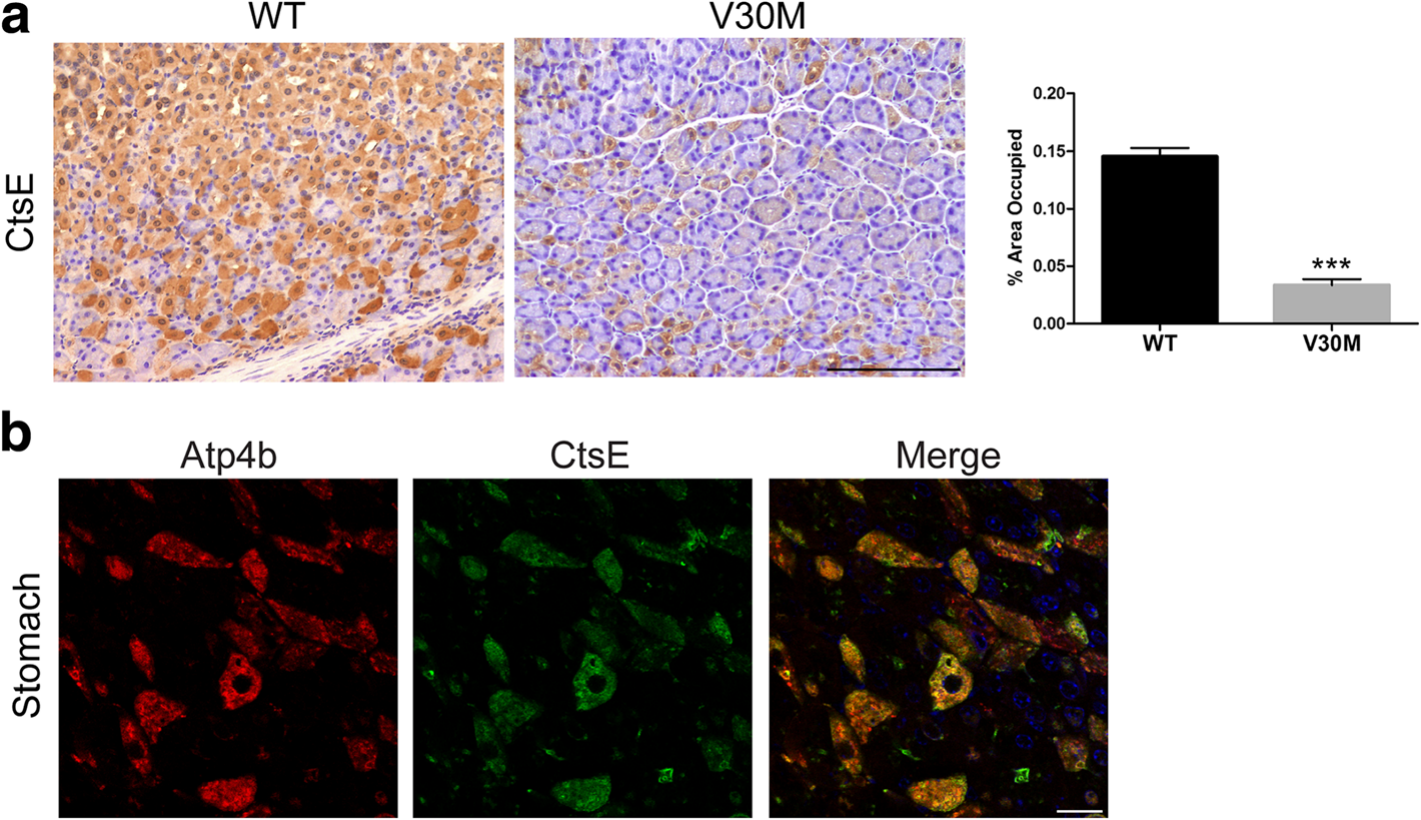
CtsE protein levels are reduced in the stomach of transgenic V30M mice. **a** CtsE protein levels in the stomach were assessed by SQ-IHQ, with respective semi-quantification of immunohistochemical images in the chart, demonstrating a downregulation in V30M mice as compared with the WT (*scale bar* 100 μm; ***p < 0.001). **b** Images obtained by confocal microscopy denoting evident colocalization between CtsE (*green*) and Atp4b (*red*) in the secretory portion of the WT mice stomach (*scale bar* 10 μm)

The gastrointestinal tract, especially the stomach, is also extensively affected by TTR V30M deposition. Therefore, CtsE levels were also assessed in this physiological system. Firstly, immunohistochemical analysis showed evident alterations in stomach protein levels of CtsE, wherein V30M mice showed a significant decrease in the protease expression, comparatively with the WT (Fig. 3a). No differences were observed regarding the esophagus, colon, or duodenum (data not shown). Moreover, double immunofluorescence between this molecule and Atp4b, a marker for parietal cells, was carried out in the stomach of WT mice. Colocalization between the markers indicates CtsE presence in the secretory portion of the stomach, more specifically in parietal cells, one of the largest epithelium cells of the mucous membrane (Fig. 3b).

### Downregulation of CtsE correlates with TTR V30M deposition

**Fig. 4:**
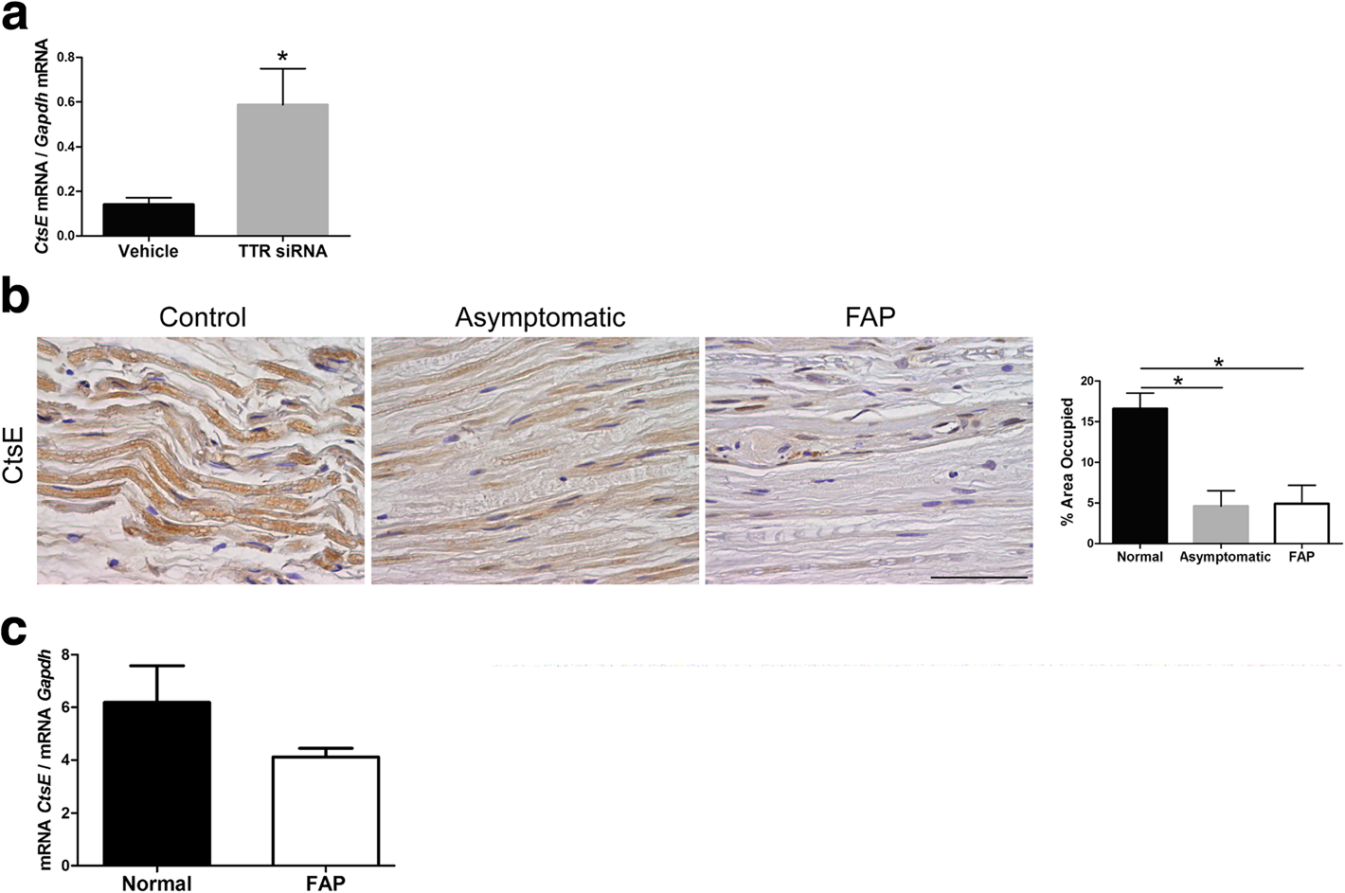
Downregulation of CtsE correlates with TTR V30M deposition. **a** Histogram represents *CtsE* mRNA levels in Hsf/V30M DRGs after mice treatment with either a TTR siRNA or vehicle (*p < 0.05). **b** CtsE immunohistochemical staining in human sural nerve biopsies, showing decreased expression of this molecule with FAP progression (*scale bar* 50 μm; *p < 0.05). **c** Real-time PCR analysis for CtsE in buffy coats from human FAP patients comparatively with healthy subjects. Patients had similar ages in both groups, and there were no gender differences. *Gapdh* was the housekeeping gene for normalization

To investigate if the reduced CtsE expression was associated with transgenic TTR deposition in the PNS, another animal model needed to be analyzed, namely we resorted to a mouse model where human TTR V30M is expressed in a *Hsf-1* null background (Hsf/V30M), resulting in TTR deposition in the PNS from 6 months of age [34]. Based on recently published data demonstrating that treatment with a TTR siRNA prevents and reverts TTR deposition in the sensory ganglia of these animals [37, 38], we treated Hsf/V30M mice with this siRNA or vehicle for 1 month. Our results point towards a correlation between decreased transcription of CtsE and transgenic TTR deposition in the DRG since inhibition of TTR deposition by siRNA rescued mRNA levels of CtsE (Fig. 4a).

Prompted by the results obtained previously with the FAP mouse models, we subsequently analyzed CtsE expression in sural nerve biopsies from FAP patients, asymptomatic carriers, and normal control subjects. With histological examination, as shown in Fig. 4b, a remarkable decrease in CtsE-stained area was observed, since the preliminary stage of disease. Thus, our data support reduced CtsE expression in mutated TTR background, both in the animal model and in FAP patients.

### CtsE as a novel potential FAP biomarker

Collectively, the results obtained so far suggest that CtsE might be a potential FAP biomarker. Ideally, disease biomarkers require an easy accessibility for diagnosis. In this sense, profiling biomarkers in the blood of patients is most useful and may allow diagnosis, prognosis, or analysis of therapeutic efficacy and progression of TTR neuropathy. Thus, we next evaluated the expression of *CTSE* by qPCR in blood samples from human FAP patients and controls. A slight reduction in the expression of CtsE was observed in FAP patients (Fig. 4c), attesting the potential of this molecule as a biomarker. Nevertheless, studies with a higher number of patient samples are still required to validate this hypothesis.

### CtsE is downregulated in bone marrow-derived macrophages

**Fig. 5:**
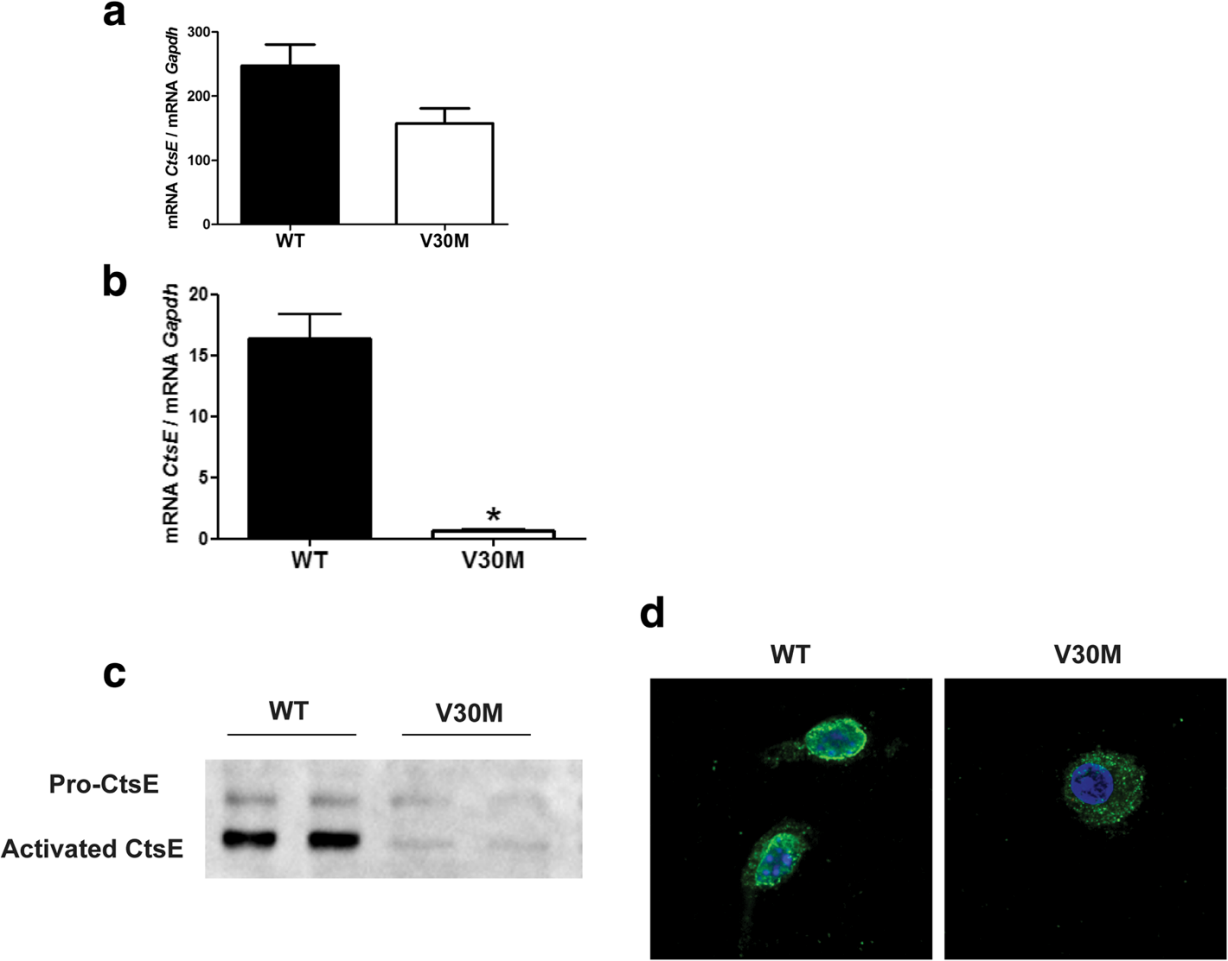
CtsE is downregulated in bone marrow and bone marrow-derived macrophages from V30M mice. Histogram represents *CtsE* mRNA levels in the **a** bone marrow and **b** bone marrow-derived macrophages from WT and V30M mice, normalized against *Gapdh*. **c** Western blots performed with macrophage lysates show both pro and activated CtsE in a much lesser extent in V30M mice. **d** Confocal microscopy denote a different staining pattern for CtsE in V30M macrophages when compared with WT (×20 magnification). Results were corroborated with three independent cell experiments

Since CtsE is highly expressed by cells of the innate immune system [26] and because blood monocytes are originated in the bone marrow, we subsequently tested the expression of CtsE in the bone marrow of V30M and WT animals. Bone marrow was isolated from the mice femur and tibia and the mixed population of cells analyzed by qPCR. In line with results in the blood, *CtsE* mRNA levels in the bone marrow collected from the transgenic mice were lower than the ones from the WT (Fig. 5a). Next, we generated bone marrow-derived macrophages for further CtsE analysis. Both at genetic and protein levels, CtsE was always found downregulated in macrophages from the V30M mice (Fig. 5b, c). Additionally, staining obtained with immunocytochemistry revealed a CtsE diffuse and less brighten pattern in V30M macrophages when compared with WT (Fig. 5d). qPCR analyses for *TTR* mRNA in macrophages did not show any message (data not shown), indicating that macrophages do not transcribe *TTR*, which means that the intracellular TTR protein found in the previous FACS analyses likely represents TTR uptake. Indeed, incubation of macrophages with oligomeric TTR V30M species indicates oligomer internalization due to its colocalization with EEA1 and Lamp-1, biochemical markers for early endosomes and lysosomes, respectively (data not shown).

In all, we show a decreased pattern of CtsE expression in bone marrow and bone marrow-derived macrophages of a pre-clinical model of TTR V30M amyloidosis. However, the TTR V30M genotype does not associate with decreased CtsE expression in all macrophages. Indeed, we tested two types of resident macrophages, obtained from the spleen or the peritoneum, and found in these cases a significant upregulation of CtsE gene expression and protein in V30M cells, as compared with those from WT mice (Additional files 1 and 2). Interestingly, both in the spleen and in peritoneal macrophages, the amount of mutated TTR was much higher than that of the WT version (Additional files 1 and 2). Thus, our results suggest that CtsE might be differentially regulated and expressed in different cellular contexts and highlight a link between increased expression of CtsE and of mutated TTR.

## Discussion

In the present study, we show impairment of CtsE in the PNS, stomach, bone marrow, and bone marrow-derived macrophages of a pre-clinical model of TTR V30M amyloidosis. It was recently described that systemic treatment with a TTR siRNA in transgenic mice is able to prevent and reverse aggregated TTR deposition in both sciatic nerve and DRGs [37, 38]. Because the transcriptional levels of CtsE in sensory ganglia were rescued upon TTR siRNA treatment, we propose that TTR aggregation in the PNS might induce a signaling cascade that culminates with transcriptional modulation of certain genes, notably of CtsE. Nerve regeneration after injury is compromised in the transgenic V30M mouse model [23], and it is well known that Schwann cell dedifferentiation and migration plays a critical role to guide and support axonal growth [39, 40]. Interestingly, Schwann cell inhibition of CtsE with pepstatin was able to impair the generation of the cell adhesion molecule L1 and consequently inhibit Schwann cell migration and myelination of sensory neurons [41]. Based on these observations and since CtsE was found considerably reduced in injured V30M nerves, it is possible that this molecule might be involved in the mechanism contributing for the lower regenerative ability present in TTR V30M amyloidosis, which is under investigation. Considering the data obtained with these mouse models of disease, we herein started exploring the potential of CtsE as a disease biomarker. Sural nerve biopsies from both symptomatic and asymptomatic V30M carriers were analyzed by histology, showing a decreased expression of CtsE. From this, we can conclude that the modulation of CtsE expression by mutated TTR is seen from very early stages of disease and is likely to require aggregates (observed in asymptomatic V30M carriers), but not amyloid deposits.

In the gastrointestinal tract, the stomach is one of the most affected organs by TTR deposition being thus extensively studied in therapeutic trials conducted on mouse models of disease [42, 43]. The decreased expression of CtsE by parietal cells was also observed in this organ, and is most likely associated with TTR V30M deposition, sharing the same genetic regulation as the one observed in the PNS.

CtsE importance in host defense mechanisms is extensively studied and known to have differential functions in different immune cell types, like dendritic cells or macrophages [44]. Macrophages are the main phagocytic cells and expected to infiltrate surrounding pre-fibrillar and TTR amyloid deposits to help on their clearance; however, in most cases, this does not happen. In fact, it was recently demonstrated that the number of heart-resident macrophages is significantly decreased in FAP patients as compared with healthy donors, with a reduced proportion of intracellular TTR in CD14+ peripheral blood monocytes, which might indeed accelerate TTR-derived amyloid deposition [45]. Furthermore, recent in vitro studies showed that when primary macrophages derived from very old Hsf/V30M transgenic mice are stimulated with TTR-aggregated toxic species, they have lower internalization and degradation rates than if pre-incubated with curcumin [46], suggesting that V30M naïve macrophages might exhibit qualitative or quantitative abnormalities. A previous study showed extensive nerve fiber loss despite small amount of amyloid deposition in late-onset FAP patients from non-endemic areas, indicating that pre-fibrillar TTR aggregates may participate in fiber degeneration in such patients [47]. In contrast, a recent study indicated that direct insult of amyloid fibrils caused Schwann cell damage, leading to nerve fiber loss, in early-onset FAP patients from endemic foci [48]. All together, these data contributes to the hypothesis that also macrophage phenotype and function may be differently modulated depending on the onset and patient endemic area. Thus, we decided to investigate transcriptional and translational levels of CtsE in bone marrow-derived macrophages. CtsE was found considerably downregulated at both mRNA and protein levels in V30M macrophages isolated from mice with 3 months of age. Macrophages with CtsE deficiency present decreased expression of surface chemotactic receptors and reduced cytokine production or antigen presentation, ultimately disrupting the overall cell functions [26, 49]. Therefore, decreased CtsE expression levels in murine bone marrow-derived macrophages, also observed in buffy coats extracted from FAP patients (although not statistically significant), can be the responsible mechanism for lower immune cell chemotaxis [23, 32] and impaired macrophage function observed in FAP [45]. Whether this is a direct or indirect effect, and which molecular players are involved, remains to be investigated. Further studies on a greater number of FAP blood samples from carriers of different TTR mutations at different ages and in different stages of disease are now critical to evaluate whether blood CtsE expression can be used as a biomarker for FAP. Moreover, samples from patients undergoing different treatments (like Tafamidis, Diflunisal, or TTR siRNA) resulting in inhibition/clearance of deposits will shed further light on the role of CtsE in FAP pathogenesis. It will also be interesting to investigate whether differences in CtsE levels in macrophages of FAP patients are then reflected in alteration of the cellular polarization profile. In particular, it will be important to study whether FAP macrophages associate more with a broad M2 phenotype, characterized by the production of anti-inflammatory cytokines and by a metabolism that relies in oxidative phosphorylation [50, 51]. This would at least in part explain the lack of inflammatory infiltrates in the nerves of FAP patients.

Macrophages lacking CtsE exhibit abnormalities in autophagy, a self-degradative system for aggregated proteins and damaged organelles. Consequently, increased accumulation of autophagy marker proteins, such as LC3 or p62, and altered autophagy-related signaling pathways end up with mitochondrial abnormalities and accompanying oxidative stress [52], programs also disrupted in FAP [17, 53]. Studies performed in human embryonic kidney and neuroblastoma cells expressing mutated TTR demonstrated that also in FAP there is a weakened autophagic response that can be modulated by curcumin and TUDCA [54, 55]. It would be very interesting to understand the autophagic flux of V30M macrophages and the possible involvement of CtsE in that process.

Given the known role of CtsE in the immune system, we also explored the expression of this molecule in different cells of the innate immune system. Interestingly, in contrast to what was observed in the bone marrow, splenic and peritoneal V30M macrophages and monocytes displayed surprising increased levels of CtsE. Thus, depending on the cell type, the impact of mutated TTR on CtsE expression is different. These results also point towards a different macrophage phenotype when located in tissues or the periphery versus in circulation or undifferentiated in the bone marrow. The mechanisms underlying these observations are not known. The observed differences may be due to differential recognition of TTR by circulating versus resident macrophages, either related to cell intrinsic properties or to amounts and conformations of TTR in different locations. It is also possible that the molecular machinery needed to regulate CtsE expression differs between these cells. Clearly, this is a complex matter that will require a combination of approaches to be fully addressed.

## Conclusions

In summary, this is the first report linking TTR V30M and CtsE expression and suggesting that mutated TTR may be involved in reprograming the transcriptional program of certain cells. It will be of importance and interest to in future investigate which reprogramming alterations occur in different cells and how mutated TTR promotes them. It will also be important to investigate what mechanisms dictate the cell-specific effects of FAP in what respects CtsE expression. In all, our findings further affirm the complex network of events underlying and being modified by amyloidosis, raising important links to disease pathogenesis. Finally, our findings also have important implications on the use of CtsE as a molecular marker of FAP.

## Additional files

Additional file 1: CtsE is overexpressed by spleen monocytes of naïve V30M mice. A) Histogram represents *CtsE* mRNA levels in the spleen of V30M mice and respective control group, normalized against *Gapdh* (*p < 0.05). B) Representative SQ-IHC against CtsE protein levels in the spleen of WT and V30M mice. Chart represents quantification of immunohistochemical images (scale bar 50 μm; *p < 0.05) C) Data represents the relative quantification of the expression of CtsE in monocytes of WT and V30M mice (**p < 0.01). D) Spleen histological examination for TTR expression in WT and V30M mice. Chart shows relative quantification of substrate positive immunoreactivity related to total tissue area (scale bar 50 μm; **p < 0.01). (TIF 23480 kb)

Additional file 2: Increased expression of CtsE and TTR in peritoneal cells of V30M mice. The frequency of macrophages, monocytes, and neutrophils expressing CtsE A) and TTR B) in the peritoneum of WT and V30M mice were analyzed by flow cytometry. Data is represented as mean ± SEM (*p < 0.05; ** *p* < 0.01). (TIF 13052 kb)

## Abbreviations

Atp4b: ATPase H+/K+ transporting beta subunit
CtsE: Cathepsin E
DRG: Dorsal root ganglia
EDTA: Ethylenediaminetetraacetic acid
EEA1: Early endosome antigen 1
FAP: Familial amyloidotic polyneuropathy
Gapdh: Glyceraldehyde 3-phosphate dehydrogenase
Hsf-1: Heat shock factor 1
IBA-1: Ionized calcium-binding adapter molecule 1
Lamp-1: Lysosomal-associated membrane protein 1
mRNA: Messenger RNA
PBS: Phosphate buffer saline
PNS: Peripheral nervous system
qPCR: Quantitative real-time PCR
SEM: Standard error of the mean
SQ-IHC: Semi-quantitative immunohistochemistry
TTR: Transthyretin
WT: Wild-type
