# Supplementary figures and images for: Differential expression of Cathepsin E in transthyretin amyloidosis: from neuropathology to the immune system
Source: J Neuroinflammation. 2017 Jun 6;14:115. doi: 10.1186/s12974-017-0891-9 (PMC5460450; doi:10.1186/s12974-017-0891-9)

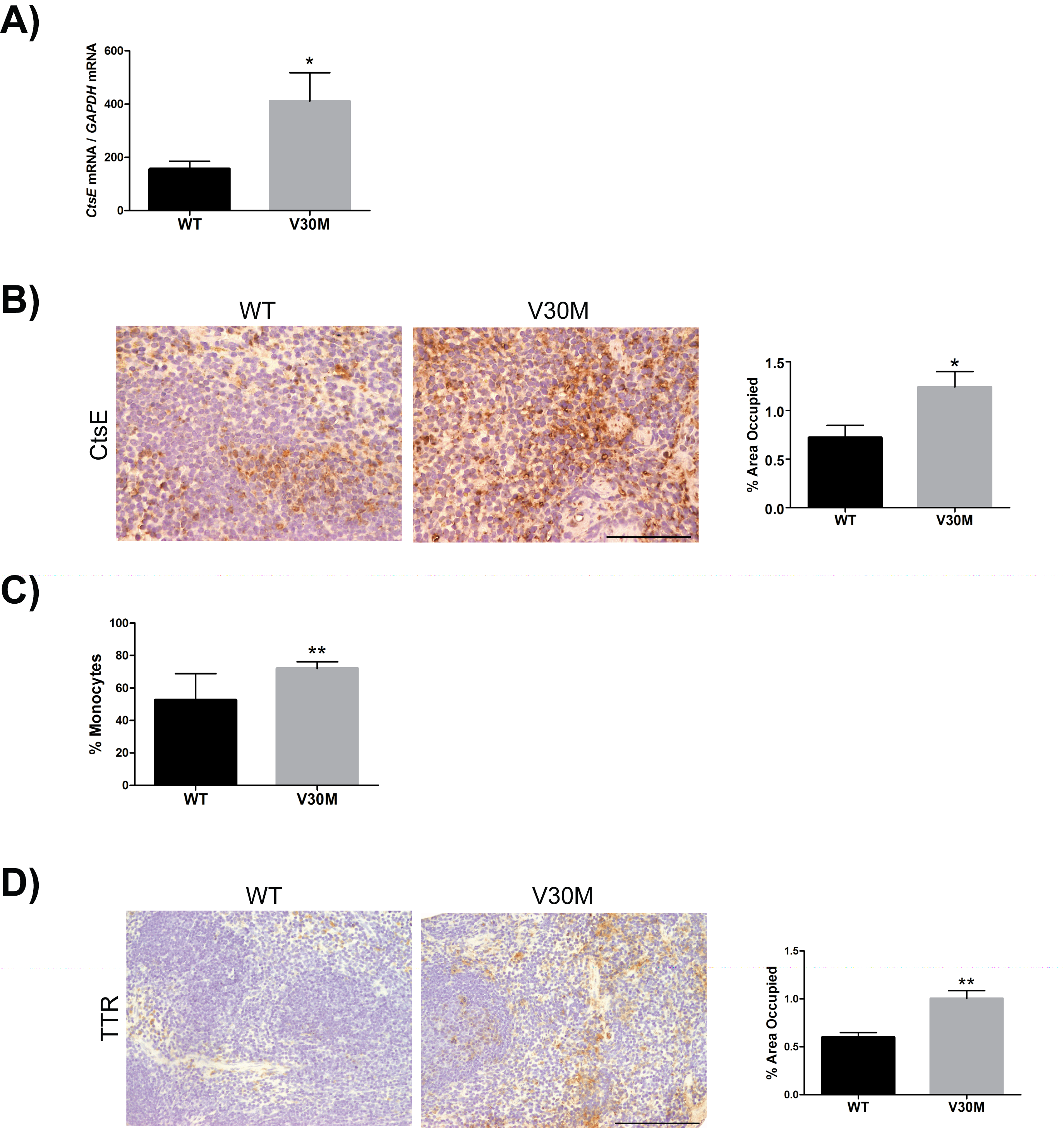

Supplement: Supplementary file 1 — CtsE is overexpressed by spleen monocytes of naïve V30M mice. A) Histogram represents CtsE mRNA levels in the spleen of V30M mice and respective control group, normalized against Gapdh (*p < 0.05). B) Representative SQ-IHC against CtsE protein levels in the spleen of WT and V30M mice. Chart represents quantification of immunohistochemical images (scale bar 50 μm; *p < 0.05) C) Data represents the relative quantification of the expression of CtsE in monocytes of WT and V30M mice (**p < 0.01). D) Spleen histological examination for TTR expression in WT and V30M mice. Chart shows relative quantification of substrate positive immunoreactivity related to total tissue area (scale bar 50 μm; **p < 0.01). (TIF 23480 kb) [file 12974_2017_891_MOESM1_ESM.tif]

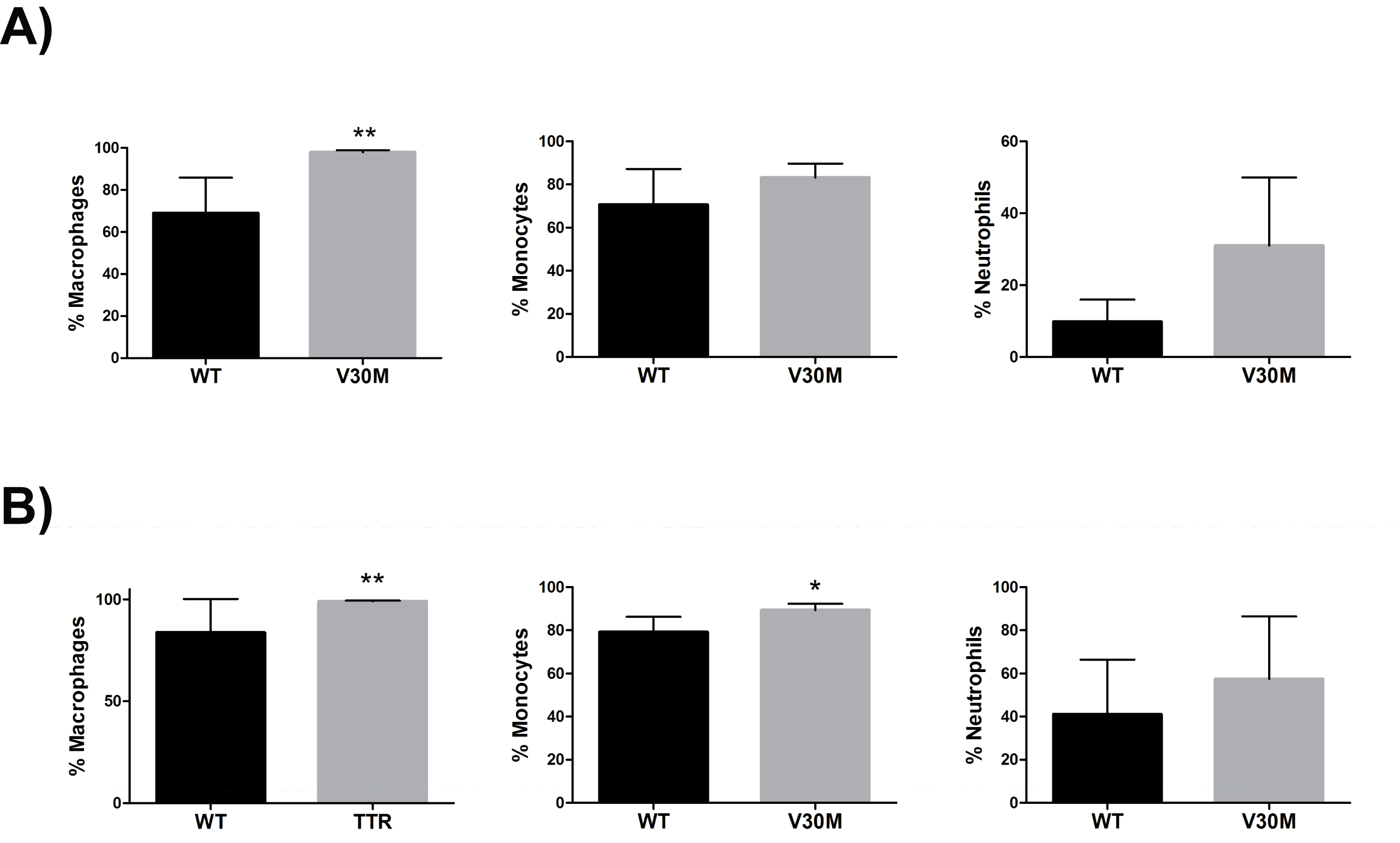

Supplement: Supplementary file 2 — Increased expression of CtsE and TTR in peritoneal cells of V30M mice. The frequency of macrophages, monocytes, and neutrophils expressing CtsE A) and TTR B) in the peritoneum of WT and V30M mice were analyzed by flow cytometry. Data is represented as mean ± SEM (*p < 0.05; ** p < 0.01). (TIF 13052 kb) [file 12974_2017_891_MOESM2_ESM.tif]
